# DE-MR simulation imaging for prone radiotherapy after breast-conserving surgery: assessing its application in lumpectomy cavity delineation based on deformable image registration

**DOI:** 10.1186/s13014-021-01817-2

**Published:** 2021-05-17

**Authors:** Changhui Zhao, Jianbin Li, Wei Wang, Guanzhong Gong, Liang Xu, Yingjie Zhang, Fengxiang Li, Qian Shao, Jinzhi Wang, Xijun Liu, Min Xu

**Affiliations:** 1 School of Medicine, Shandong University, Jinan, 250012 Shandong Province China; 2 Department of Radiation Oncology, Shandong Cancer Hospital and Institute, Shandong First Medical University and Shandong Academy of Medical Sciences, 440 Jiyan Road, Jinan, 250117 Shandong Province China; 3 Department of Medical Physics, Shandong Cancer Hospital and Institute, Shandong First Medical University and Shandong Academy of Medical Sciences, Jinan, 250117 Shandong Province China; 4 Department of Medical Imagings, Shandong Cancer Hospital and Institute, Shandong First Medical University and Shandong Academy of Medical Sciences, Jinan, 250117 China

**Keywords:** Prone radiotherapy, Breast-conserving surgery, Lumpectomy cavity delineation, Computed tomography simulation image, Delayed-enhancement magnetic resonance simulation image

## Abstract

**Background:**

The application of delayed-enhancement magnetic resonance (DE-MR) simulation imaging in lumpectomy cavity (LC) delineation for prone radiotherapy in patients with an invisible seroma or a low seroma clarity score (SCS) after breast-conserving surgery (BCS) based on deformable image registration (DIR) was assessed.

**Methods:**

Twenty-six patients who were suitable for radiotherapy in prone positions after BCS were enrolled, and both computed tomography (CT) and DE-MR simulation scans were acquired. The LC delineated based on titanium surgical clips on CT images was denoted as LC_CT_. The LC delineated based on the signal of cavity boundaries on fat-suppressed T2-weighted imaging (T2WI) and multiphase delayed-enhancement T1-weighted imaging (DE-T1WI), which was performed at 2 min, 5 min and 10 min postinjection, were denoted as LC_T2_, LC_2T1_, LC_5T1_ and LC_10T1_, respectively. Afterwards, DIR was performed to compare the volumes and locations of the LCs with MIM software. The generalized conformity index (CIgen) of inter (intra) observer (Inter-CIgen and Intra-CIgen) was also used to explore the inter(intra) observer variation for LC delineation on each image modality.

**Results:**

LC_CT_–LC_10T1_ provided the best conformal index (CI) and degree of inclusion (DI), increasing by 2.08% and 4.48% compared to LC_CT_–LC_T2_, 11.36% and 2.94% for LC_CT_–LC_2T1_, and 8.89% and 7.69% for LC_5T1_–LC_CT_, respectively. The center of mass (COM) of LC_CT_–LC_10T1_ decreased by 17.86%, 6.12% and 13.21% compared with that of LC_CT_–LC_T2_, LC_CT_–LC_2T1_ and LC_CT_–LC_5T1_, respectively. The agreement of LC delineation was strongest for 10th min DE-TIWI (coefficient of variation, COV = 2.30%, Inter-CIgen = 87.06%, Intra-CIgen = 92.64%).

**Conclusion:**

For patients with a low SCS (SCS ≤ 2) after BCS, it is feasible to contour the LC based on prone DE-MR simulation images. Furthermore, the LC derived from prone DE-T1WI at 10 min was found to be most similar to that derived from prone CT simulation scans using titanium surgical clips regardless of the volume and location of the LC. Inter (intra) variability was minimal for the delineation of the LC based on 10th min DE-TIWI.

## Background

Breast-conserving therapy (BCT) has been offered as the standard care for patients with early breast cancer [1–3]. Adjuvant radiotherapy (RT), such as whole breast irradiation (WBI) with an additional boost delivered to the lumpectomy cavity (LC) or partial breast irradiation (PBI), is an important component in BCT, as it reduces locoregional recurrence (LRR) and improves overall survival (OS) [4, 5]. Given that adjuvant RT often delivers a therapeutic radiation dose to the clinical target volume and that radiation morbidity is directly related to the irradiated volume, an accurate delineation of the LC is a prerequisite to achieve treatment efficiency and to decrease acute/late toxicity.

To date, as the standard reference imaging modality, computed tomography (CT) simulation imaging has been used to localize the LC [6, 7]. Both titanium surgical clips and seromas are important markers for delineating the LC based on CT simulation images [8, 9]]. Many previous studies have advocated that various landmarks, such as the number and location of titanium surgical clips and the seroma clarity score (SCS) [10], within the excision cavity can influence the accuracy of LC delineation [7, 8, 12]. According to previous studies, interobserver variation decreases significantly as the SCS increases, and variability is lowest in patients with an SCS of 3–5 [11, 12]. When the SCS is equal to or greater than 3, observer consistency in LC contouring can be improved when the number of surgical clips is 5–6 [13]. However, the accuracy of an SCS < 3 to mark LCs remains controversial, as seroma visibility is too low for observers to distinguish.

Given the lack of contrast observed on CT images, several investigators have proposed the use of additional image-guided techniques. On account of the intrinsically high soft tissue contrast of magnetic resonance imaging (MRI), LC can be better identified, hence making it a promising tool in breast RT simulation [14]. When seroma is visible, noncontrast MRI also improves the LC SCS, interobserver concordance and accuracy for patients without clips in the LC compared to CT simulation imaging [15–17]. If the SCS is too low to be determined, it seems that no valid information can be obtained, even from CT and noncontrast MR coregistered images [18, 19].

Several studies have shown that LCs can be identified easily on delayed-enhancement MRI (DE-MRI) for patients with SCS > 3 [20, 21]. Thus, we compared prone CT simulation images and different sequences of prone DE-MR simulation images for LC delineation in patients whose excision cavity had a low SCS but an appropriate number of titanium surgical clips after breast-conserving surgery (BCS). The better time for acquiring DE-MR simulation images in LC delineation was also analyzed.

## Materials and methods

### Patient selection

Patients with early-stage breast cancer (pT1-2; N0; M0) who were treated with BCS were included in our study. The characteristics of the 26 patients studied are listed in Table 1. All patients were suitable for prone RT based on body condition, breast size and LC position. All patients underwent lumpectomy with 5–6 titanium surgical clips implanted superior, inferior, medial, lateral, and posterior to the LCs, and when simulated, the SCS in the surgical cavity was less than or equal to 2. Patients with contraindications for MRI or oncoplastic BCS were excluded, and it was necessary for all patients to cooperate well with breathing training. Written informed consent was obtained from all enrolled patients who voluntarily underwent postoperative DE-MR and CT simulation scans in the prone position. This study was approved by the Institutional Review Board of the Shandong Cancer Hospital and Institute Ethics Committee (SDTHEC201703014).

**Table 1:** Characteristics of 26 patients studied

Characteristics	No. of cases (%)
Age (y), median (range)	45 (29–53)
Breast side	
Left	12 (46.15)
Right	14 (53.85)
Tumor Location	
OUQ	12 (46.15)
OLQ	2 (7.69)
IUQ	7 (26.92)
ILQ	0 ( 0.00)
Central portion of breast	5 (19.23)
Stage	
T1b	7 (26.92)
T1c	16 (61.54)
T2	3 (11.54)
Pathologic type	
IDC	20 (76.92)
DCIS	6 (23.08)
SCS	
0	16 (61.54)
1	7 (26.92)
2	3 (11.54)
No. of titanium surgical clips	
5	17 (65.38)
6	9 (34.62)
Time interval from surgery to planning CT scan (days), median (range)	122 (30–198)

*OUQ* outer upper quadrant, *OLQ* outer lower quadrant, *IUQ* inter upper quadrant, *ILQ* inter lower quadrant, *DCIS* Ductal carcinoma in situ, *IDC* Invasive ductal carcinoma, *SCS* seroma clarity score

### Image acquisition

**Fig. 1:**
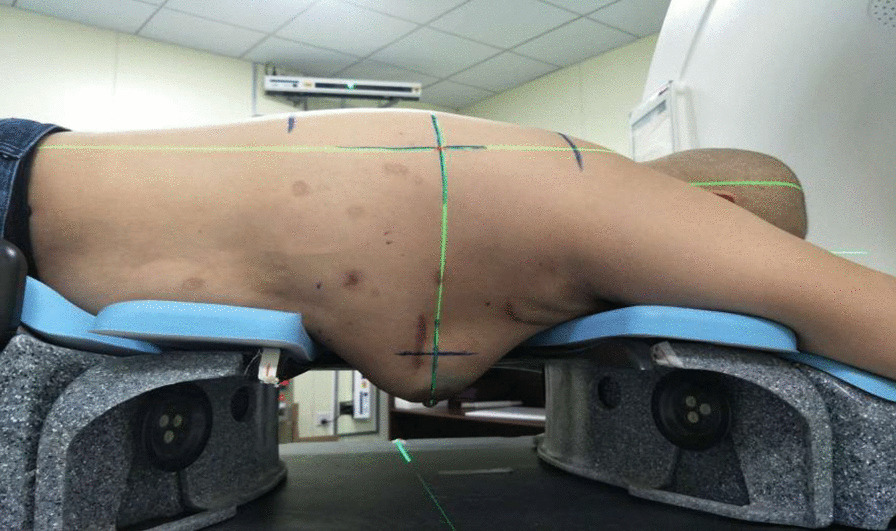
Prone treatment board and placement of markers before simulation scanning

Patients underwent postoperative prone CT simulation scans (Philips Medical Systems, Inc., Cleveland, OH) on a patient-specific treatment board (CIVCO Horizon™ Prone Breast Bracket, MTHPBB01) with both arms above the head (Fig. 1). The contralateral breast was abducted adequately, while the treated breast was hung freely away from the chest wall through an opening in the board. As the marks on the ipsilateral breast, back and side were aligned with lasers, noncontrast CT simulation scans were acquired.

Acquired immediately after or on the same day as CT simulation scans, the MR simulation scans were collected with a specially designed 32-element phased-array breast coil by a 3.0-T, 70-cm bore MR scanner (750 W, General Electric Co., Boston, USA). During MR simulation scans, the patients were immobilized with the same dedicated device and in the same position as in CT simulation scans. A total of 4 pulse sequences of MR simulation images were acquired in turn. First, fat-suppressed T2W images with the inhibition of motion artifacts were acquired with patients under free breathing. This was followed by multiphase delayed-enhancement T1-weighted imaging (DE-T1WI) of the ipsilateral breast with fat suppression, performed at 2 min, 5 min and 10 min postcontrast subtraction with patients under breath holding. The characteristics of all pulse sequences used in this study are summarized in Table 2. All enhanced sequences were injected with 15 mL of contrast agent (gadopentetate dimeglumine) at 2 mL/s. Afterwards, 20 ml of normal saline was injected to ensure that the contrast agent was fully absorbed into the body.

**Table 2:** Parameters of MR simulation pulse sequences

	T2WI	2nd min DE-TIWI	5th min DE-TIWI	10th min DE-TIWI
TR/TE (ms)	7059/81	4.7/2	4.7/2	4.7/2
Slice thickness (mm)	3	3	3	3
FOV (mm)	420	420	420	420
Acquisition time (s)	450	18	18	18
Acquisition matrix	256 × 292	256 × 292	256 × 292	256 × 292

*TR *time of repeatation, *TE* time of echo, *FOV* field of view

The slice thickness of both the CT and MR simulation images was 3 mm, and all images were transferred to MIM version 6.8.3 software (Cleveland, USA).

### LC delineation

**Fig. 2:**
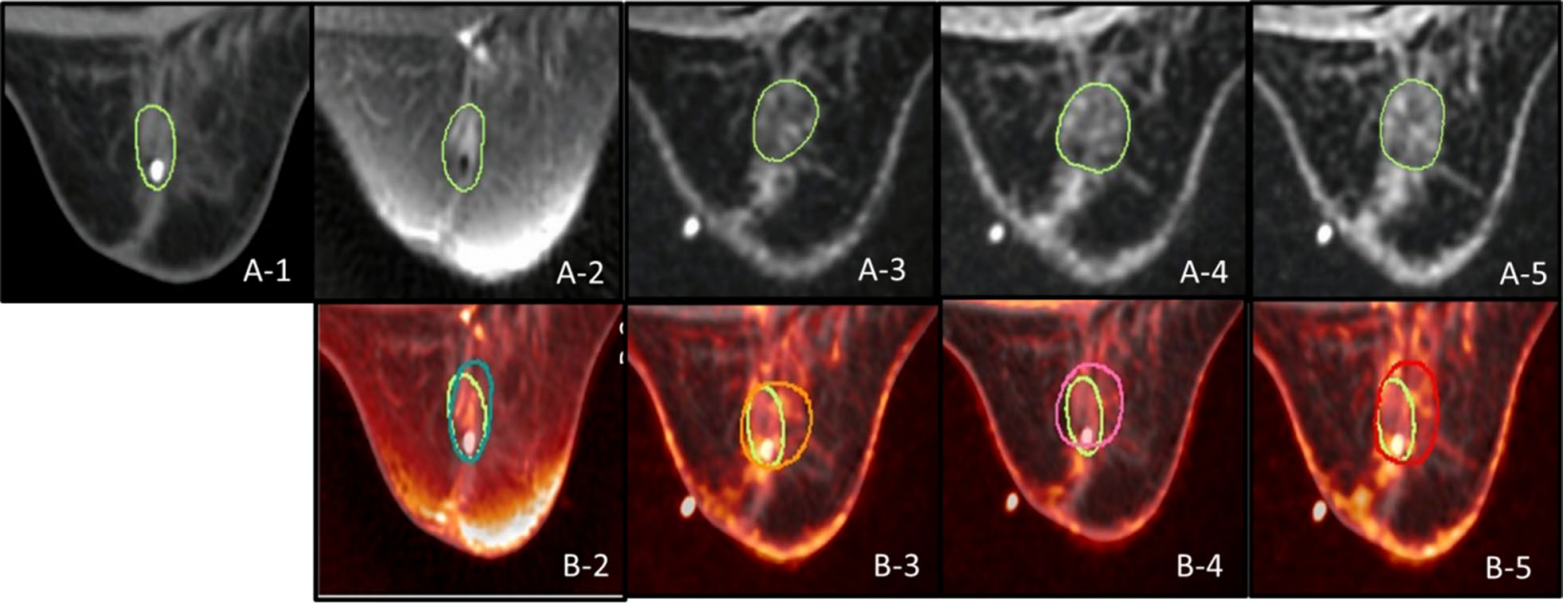
Contours of LC and the comparison of LC on fusion images between different sequences of MRI and CT scans (a-1: LC contours delineated on CT simulation axial images based on titanium surgical clips; a-2–5:LC contours delineated on T2WI, 2nd min DE-TIWI, 5th min DE-TIWI and 10th min DE-TIWI based on the signal of cavity boundaries. b-2–5: LC contours delineated on the fusion of T2WI, 2nd min DE-TIWI, 5th min DE-TIWI and 10th min DE-TIWI to CT simulation axial images, respectively)

The LCs were manually delineated on CT and MR simulation images by three experienced radiation oncologist. The LCs derived from CT simulation images were based only on the placement of the titanium surgical clips and were defined as LC_CT_ (Fig. 2a1). On T2WI with fat suppression and on DE-T1WI at 2 min, 5 min or 10 min, the LCs were delineated based on the visible MR signal of the surgical cavity and defined as LC_T2_, LC_2T1_, LC_5T1_ and LC_10T1_ (Fig. 2a2-5). The LC contours delineated on the fusion of T2WI, 2nd min DE-TIWI, 5th min DE-TIWI and 10th min DE-TIWI to CT simulation images were shown in Fig. 2b2-5. The interval of the delineation of LC_T2_, LC_2T1_, LC_5T1_ and LC_10T1_ was 2 weeks. To avoid providing a reference for the new LCs, the LCs that had been contoured were not shown when contouring the new LCs. The time required for LC delineation was also recorded.

### Deformable image registration (DIR) procedure

The DIR procedure of CT and MR simulation images consisted of 4 consecutive steps that were implemented using the MIM system. The time taken for DIR was approximately 3 to 5 min per patient. During the DIR procedure in this study, prone CT simulation images represented the main sequence, and prone MR simulation images represented the subordinate sequence. Afterwards, according to the workflow in MIM, the user performed an automatic rigid registration between the CT simulation images and each sequence of the MR simulation images. As rigid registration was approved, DIR was used to resample the MRI data for fusion with the CT data for each patient separately. Finally, based on automatic deformation, the Reg Reveal tool was used for evaluating DIR in the primary area of concern [22]. Reg Refine would only be used in the event that, while evaluating the initial deformation with Reg Reveal, it was determined a poor alignment was identified that needs to be fixed [23]. Converting local alignments, defined as an assemblage of local alignments to create a deformable registration, was used in our study. Points of skin, nipple, sternum and ribs were locked by Reg Refine to guarantee better registration of the surgical cavity and treated breast. Then, they were combined into an overall deformable registration after rigid registration was approved. Note that a Gaussian mixing model was used in this combination to spatially weight the contributions of each local rigid alignment. Eventually, the point contours were regarded as a reference by DIR quality assurance (QA) to see how close these markers came to matching after the DIR was ran.

### Parameter evaluation

After DIR was completed, not only the volumes of the LCs but also the three-dimensional (3D) coordinates of all the geometric centers of the LCs were assessed with MIM software. The displacements (i.e., the differences between the maximum coordinates and minimum coordinates) between the structures contoured on the CT and MR simulation images in the lateral (LR), anteroposterior (AP) and superoinferior (SI) directions were obtained and defined as Δx, Δy and Δz, respectively. All the approaches that provided a useful assessment of LC volumes were categorized into the following groups: (1) simple LC volume analysis; (2) center of mass (${\text{COM = }}\sqrt {\Delta {\text{x}}^{2}+\Delta{\text{y}}^{2}+\Delta{\text{z}}^{2} }$); (3) conformal index (CI, CI = (A ∩ B)/(A ∪ B)) and degree of inclusion (DI, DI = (A ∩ B)/A); (4) The generalized conformity index (CIgen, CIgen = $\sum\nolimits_{pairs\,i\,j} {\left| {A_{i} \cap A_{j} } \right|} /\sum\nolimits_{pairs\,i\,j} {\left| {A_{i} \cup A_{j} } \right|}$), defined as ratio of all overlapping volumes between pairs of observers and the sum over all observer pairs of their encompassing volumes (delineated by at least one observer), and the coefficient of variation (COV, COV = standard deviation/ mean), were used to analyze the inter- and intraobserver variation (Inter-CIgen and Intra-CIgen) for LC countering on each image modality. Subsequently, the information obtained from MIM software was calculated based on the formulas as described previously. In general, the CI and DI ranged from 0 to 1, where 1 represents total unity between volumes and 0 represents disunity between volumes. Note that CIgen ranges between 0 (no concordance) and 1 (100% concordance). In addition, we allocated the patients into two groups according to the breast volume size to analyse the influence of the breast volume size on the parameters of the targets. Based on the definition reported by Kim et al. [24], the patients with 550 cm^3^ or over sized breast were defined as large breast volume group and less than 550 cm^3^ as small breast volume group in this study.

### Statistical analysis

The Wilcoxon signed-rank test was used to compare the volume or delineation time of LCs (LC_CT_ versus LC_T2_, LC_2T1_, LC_5T1_ or LC_10T1_) since they did not follow a normal distribution. One-way analysis of variance (ANOVA) was used to compare differences in parameters such as the CI, DI and COM between the CT and MRI cohorts, as was inter (intra) observer variability for LC delineation on different image modalities. The relevance of differences between LC volumes was calculated by Spearman rank correlation analysis. Mann–Whitney-U-test was applied to analyse the variability between large breast volumes and small ones. Statistical analysis was performed using SPSS 19.0 software (IBM Corporation, Armonk, NY, USA). A *P* value < 0.05 was considered significant.

## Results

Between September 2018 and July 2019, 26 patients were enrolled in this study, and the median patient age was 45 years (range, 29–53 years). Of the 26 patients 76.92% were diagnosed with ductal carcinoma in situ (DCIS), and the other 23.08% were diagnosed with invasive ductal carcinoma (IDC). All patients underwent a lumpectomy and were confirmed to have negative tumor margins during the single operation. The SCS values on CT simulation images varied from 0 to 2 for the patients studied (median, 0).

### The inter(intra) observer variation for LC delineation

The inter- and intraobserver variation (Inter-CIgen and Intra-CIgen) for LC on each image modality are listed in Table 3. The agreement of LC delineation was strongest for 10th min DE-TIWI(COV = 2.30%, Inter-CIgen = 87.06%, Intra-CIgen = 92.64%) followed by T2WI (COV = 5.45%, Inter-CIgen = 83.69%, Intra-CIgen = 92.24%) and the agreement of LC delineation was lowest for CT (COV = 8.97%, Inter-CIgen = 73.88%, Intra-CIgen = 86.83%). The differences among each image modality for Inter-CIgen and Intra-CIgen did not reach statistical significance (all *P* > 0.05).

**Table 3:** _Inter- and intra-observer COV and CIgen for LC volumes (%, Mean)_

Image modality	COV	Inter-CIgen	Intra-CIgen
CT	8.97	73.88	86.83
T2WI	5.45	83.69	92.24
2nd min DE-TIWI	8.20	82.52	91.22
5th min DE-TIWI	8.48	81.91	90.21
10th min DE-TIWI	2.30	87.06	92.64

*COV* coefficient of variation, *CIgen* generalized conformity index

### Comparison of the delineation times

**Fig. 3:**
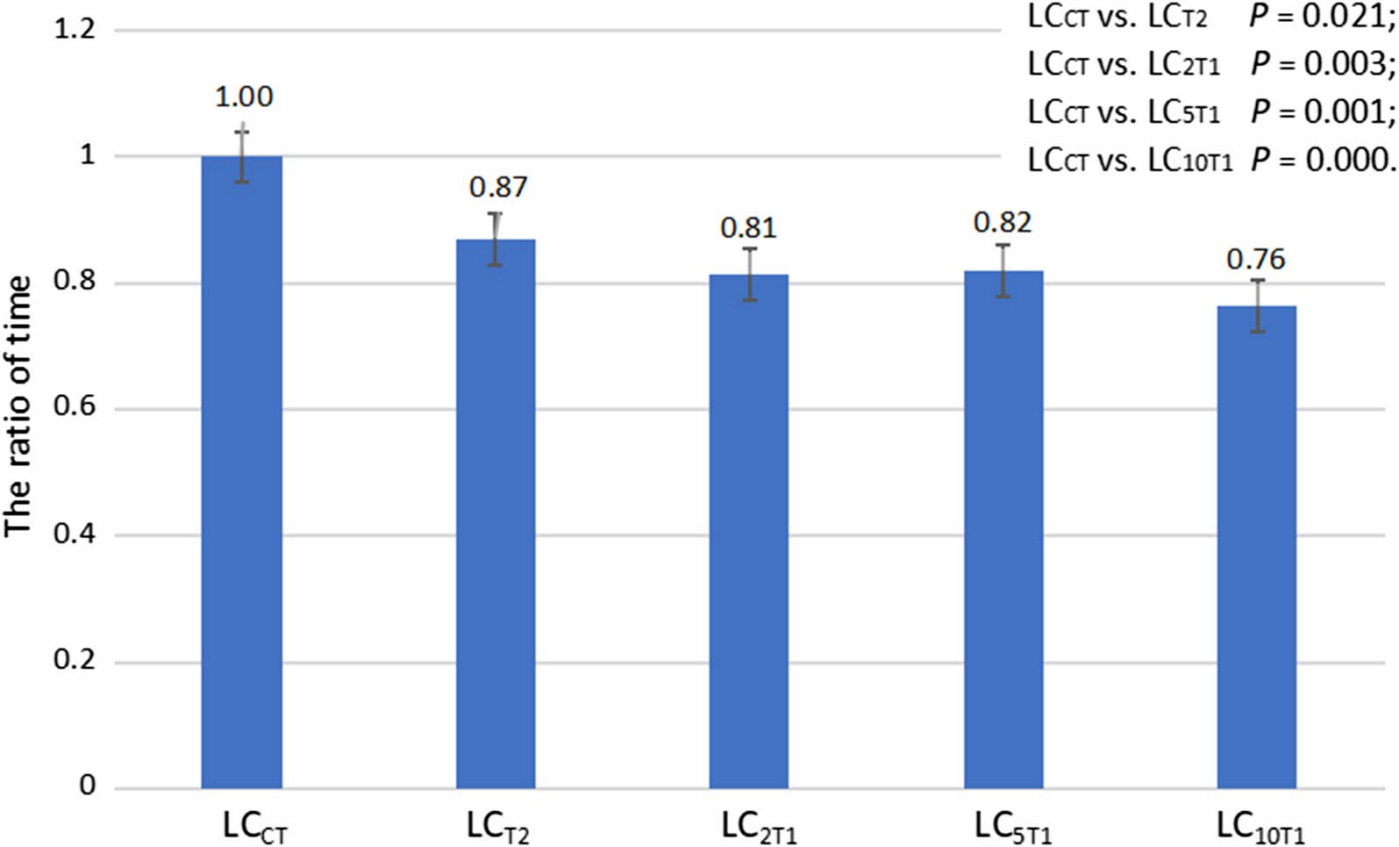
The ratio of time required to delineate the LCs based on prone CT simulation images and various sequences of prone MR simulation images

The time required to delineate LC_T2_, LC_2T1_, LC_5T1_ and LC_10T1_ accounted for 86.96%, 81.30%, 81.97% and 76.34%, respectively, of that required to delineate LC_CT_ (*P* = 0.021, 0.003, 0.001, and 0.000, respectively) (Fig. 3). Furthermore, the time required to contour LC_CT_ and LC_10T1_ showed the largest difference, with a median ratio of 1.31 (*Z* = 3.516, *P* = 0.000).

### Comparison of the LC volumes and correlation analysis

The LC_CT_, LC_T2_, LC_2T1_, LC_5T1_ and LC_10T1_ volumes are listed in Table 4. The LC_2T1_ and LC_5T1_ volumes were 2.20 cm^3^ and 1.49 cm^3^ larger than the LC_CT_ volume, respectively (*Z* = − 2.914 and − 2.601, respectively; *P* = 0.004 and 0.009, respectively). However, there was no statistically significant difference between the LC_CT_ volume and the LC_10T1_ or LC_T2_ volume (*Z* = − 1.810 and − 1.855, respectively; *P* = 0.064 and 0.070, respectively). The LC_CT_ volume was proven to be significantly positively correlated with those of LC_T2_, LC_2T1_, LC_5T1_ and LC_10T1_ (*r* = 0.904, 0.852, 0.888, and 0.929, respectively, all *P* < 0.05).

**Table 4:** LC volumes delineated based on prone CT simulation images and different prone MR simulation images (cm^3^)

LC volume	Median (cm^3^)	Range (cm^3^)	*Z* value	*P* value
LC_CT_	9.96	6.44–17.09		
LC_T2_	12.28	7.26–17.59	− 1.855	0.064
LC_2T1_	12.16	8.41–20.80	− 2.914	0.004
LC_5T1_	11.45	8.23–19.56	− 2.601	0.009
LC_10T1_	14.62	7.30–20.64	− 1.810	0.070

*LC*_*CT*_ the LC delineated based on CT simulation images, *LC*_*T2*_ the LC delineated based on T2WI, *LC*_*2T1*_ the LC delineated based on 2nd min DE-TIWI, *LC*_*5T1*_ the LC delineated based on 5th min DE-TIWI, *LC*_*10T1*_ the LC delineated based on 10th min DE-TIWI

### LC comparison

The comparisons of the image registration results are shown in Table 5. When considering the CI, DI and COM, we found that LC_CT_–LC_10T1_ was better than other sequences, although there were no statistically significant differences between them (*F* = 0.580, 0.628 and 0.935, respectively; *P* = 0.584, 0.661 and 0.432, respectively). It was noted that compared to LC_CT_–LC_T2_, LC_CT_–LC_2T1_ and LC_CT_–LC_5T1_, the CI and DI were improved by LC_CT_–LC_10T1_. They increased by 2.08% and 4.48% for LC_CT_–LC_T2_, 11.36% and 2.94% for LC_CT_–LC_2T1_, and 8.89% and 7.69% for LC_CT_–LC_5T1_, respectively. For all patients in our study, the COM of LC_CT_–LC_10T1_ decreased by 17.86%, 6.12% and 13.21% compared with that of LC_CT_–LC_T2_, LC_CT_–LC_2T1_ and LC_CT_–LC_5T1_, respectively.

**Table 5:** Parameter evaluation of the LC defined using prone CT simulation images and different prone MR simulation images based on DIR

	LC_CT_–LC_T2_		LC_CT_–LC_2T1_		LC_CT_–LC_5T1_		LC_CT_–LC_10T1_		*F* value	*P* value
	Median	Range	Median	Range	Median	Range	Median	Range		
CI	0.48	0.35–0.56	0.44	0.39–0.58	0.45	0.30–0.55	0.49	0.38–0.56	0.584	0.628
DI	0.67	0.57–0.75	0.68	0.60–0.84	0.65	0.56–0.79	0.7	0.56–0.85	0.661	0.58
COM (cm)	0.56	0.40–0.73	0.49	0.38–0.70	0.53	0.37–0.70	0.46	0.31–0.80	0.935	0.432

*CI* conformal index, *DI* degree of inclusion, *COM* the distance between the center of mass of the targets, *LC*_*CT*_ the LC delineated based on CT simulation images, *LC*_*T2*_ the LC delineated based on T2WI, *LC*_*2T1*_ the LC delineated based on 2nd min DE-TIWI, *LC*_*5T1*_ the LC delineated based on 5th min DE-TIWI, *LC*_*10T1*_ the LC delineated based on 10th min DE-TIWI

### Difference of parameters between large and small breast size

The variability between large breast volumes and small ones are shown in Table 6. The patients with 550 cm^3^ or over sized breast were defined as large ones, accounting for 69.23% in our study. No significant difference was found between the two groups either in the delineation time or in the parameters (all *P* < 0.05).

**Table 6:** Difference of the parameters between the different breast volume groups

	LC_CT_–LC_T2_ (Median)			LC_CT_–LC_2T1_ (Median)			LC_CT_–LC_5T1_ (Median)			LC_CT_–LC_10T1_ (Median)		
breast volume (cm^3^)	CI	DI	COM (cm)	CI	DI	COM (cm)	CI	DI	COM (cm)	CI	DI	COM (cm)
≥ 550	0.461	0.617	0.428	0.425	0.678	0.432	0.464	0.600	0.601	0.506	0.670	0.339
< 550	0.410	0.646	0.577	0.441	0.711	0.559	0.449	0.695	0.539	0.425	0.667	0.517
*P*	0.728	0.810	0.979	0.437	0.936	0.437	0.611	0.437	0.769	0.679	0.953	0.513

*CI* conformal index, *DI* degree of inclusion, *COM* the distance between the center of mass of the targets, *LC*_*CT*_ the LC delineated based on CT simulation images, *LC*_*T2*_ the LC delineated based on T2WI *LC*_*2T1*_ the LC delineated based on 2nd min DE-TIWI, *LC*_*5T1*_ the LC delineated based on 5th min DE-TIWI, *LC*_*10T1*_ the LC delineated based on 10th min DE-TIWI

## Discussion

Supine breast radiotherapy represents the common approach after BCS for most breast cancer patients. While the interests in the development of treatment strategies with prone breast radiotherapy has increased, and it maybe become the preference for partially appropriate breast cancer patients [25, 26]. For adjuvant radiotherapy after BCS, the accuracy of LC is crucial for both supine and prone positioning RT. The current gold standard of LC delineation is using standardized guidelines coupled with CT/seroma and surgical clips when present [8, 27]. However, either seroma or surgical clip has its own limitations in LC contouring, for example, the seroma volume and SCS decrease over time, cases with or without an insufficient number of surgical clips in the excision cavity, and architectural distortion caused by oncoplastic surgical techniques lead to the inconsistency between surgical clips and primary tumor location [28–34]. Therefore, in our study, all patients were implanted with 5–6 titanium surgical clips in the cavity, as this is considered the optimal number of markers in BCT [13]. To facilitate the comparison, LC_CT_ delineated based on titanium surgical clips on the CT simulation image was regarded as the reference target in this study.

Until now the advantages of DE-MRI in identifying LC have been shown in several studies [20, 21]. The inter (intra)observer variation for LC delineation on CT and each MRI image modality all showed no significant difference. However, DE-MR and fat-suppressed T2WI yielded better inter(intra)observer variation than CT scans. The concordance of LC delineation was strongest for 10th min DE-TIWI (COV = 2.30%, Inter-CIgen = 87.06%, Intra-CIgen = 92.64%). The Dice coefficient is an effective method to evaluate the performance of the DIR. Previous studies found that the Dice coefficient produced by DIR was 0.65 for CT/MRI and 0.43 for CT/PET-CT [35, 36]. In our study, the Dice coefficient of 0.7 increased by approximately 7.14% or 38.57% compared with other reports. Hence, we explored the best MRI-simulation scanning sequences and the best delayed time further for delineating the LC.

Several imaging modalities, including MRI, ultrasound (US), and positron emission tomography (PET) CT, have been explored to improved the accuracy of LC delineation, but MRI has shown to be superior due to its soft tissue contrast [14, 15, 17]. Our results reveal that when patients have an invisible seroma or an inferior SCS, LCs can be distinguished more easily on both fat-suppressed T2WI and fat-suppressed DE-T1WI than on CT simulation images. But noncontrast, nonfat-suppressed MRI does not improve the interobserver concordance of LC delineation compared to CT images even for patients with surgical clips and high SCS [18, 19]. Concerning patients who underwent open cavity surgical techniques with either no surgical clips or poor seroma clarity, Jolicoeur et al. found that interobserver variability generated from T2WI without fat suppression was smaller than that generated from noncontrast CT images for LC delineation [15]. As shown in Table 3, the inter-CIgen obtained on MR was better than that derived from CT images, implying that the volume and location of the LC achieved better concordance among the three observers based on MR than CT images. This discrepancy may be due to the better LC contrast with normol breast soft tissue of MRI than CT, the various surgical techniques (open- and closed-cavity surgical technique) and so on.

A postoperative complex, which includes seroma contains mixed fat and minimal water signal, and the cavity wall acts as a surrogate for the LC on postoperative MR simulation images [37]. Previous studies of postoperative MRI have demonstrated correlations between the signal characteristics of nonfat-suppressed T2WI and cavity contents, such as seromas [15]. However, the cavity wall, formed by granulation tissue, is difficult to detect on nonfat-suppressed T2WI. In a study by DEN et al. [38], patients with inferior visibility of LC potentially benefited from the use of fat-suppressed T2WI, since there was clear contrast between seroma and fibroglandular tissue. We contoured LC_T2_ (Fig. 2) on fat-suppressed T2WI, as the patients recruited were without a seroma or with a poor SCS (≤ 2). Although no significant difference between the volume of LC_T2_ and that of LC_CT_ was found, the CI and DI between LC_T2_ and LC_CT_ were only 0.48 and 0.67, respectively, indicating that the shapes of the contours being different. When delineating the LC on fat-suppressed T2WI, close attention should be paid to patients long after surgery who with lower SCS, in which a low cavity wall signal might be the result of the evolution of granulation tissue into fibrous tissue (Fig. 2A2; SCS = 0). However, the time limit remains unclear. The high LC signal remained on fat-suppressed T2WI even though the longest time from surgery in our study was 198 days. We also found the parameter evaluation of LCs, defined using prone CT simulation images and different prone MR simulation images, had nothing to do with the breast volumes.

Enhancement can be homogeneous or heterogeneous which may be associated with fat signal intensity, fat necrosis, signal voids, or resolving edema, so breath holding-DE-T1WI acquired by an MR scanner can provide superior soft tissue contrast [20, 39, 40]. Hence, we innovatively regard breath holding DE-T1WI as simulation scans for breast cancer patients who underwent prone RT. To explore which DE time points were better in LC delineation for patients with an invisible seroma or a poor SCS, for the first time, we obtained multiphase breath holding-DE-T1WI. It was noted that the enhancement surrounding the DE-T1WI excision cavity progressively increased over time, and LC_10T1_ yielded maximal enhancement. LC_10T1_ was better than other DE time points or T2 in terms of correlations with the LC volume and location. LC_CT_–LC_10T1_ also offered better spatial overlap than the other DE-T1WI sequences across all patients. LC enhances on contrast MRI is the result of pathophysiological reactions to wound repair, including inflammatory infiltration, granulation tissue proliferation, and the increasing number and permeability of the vasculature. Owing to the structural characteristics of vascularized granulation tissue, contrast material will accumulate at the pericavity during the delayed phase [14, 41]. Among our patients, the median interval after BCS was 122 days, during which the granulation tissue formation might have evolved into fibrous tissue during wound healing. As a result, the granulation tissue where most contrast material flowed in and out (blood clotting, inflammation, and finally tissue remodeling) slowly showed persistent enhancement over time on DE-MRI, and of course, LC_10T1_ had the highest signal around the LC in our study.

Compared with the previous study of LC contouring on MRI, a new scanning sequence (breath holding-DE-MRI) and multiperiod scanning were applied in our study [18–35]. Though breathing control can decrease respiratory movement-associated artifacts, our results showed that LC_CT_ was smaller than the LC derived from MRI regardless of the scanning sequence used. Breath holding-DE-MRI could provide additional information for LC contouring when compared to CT coupled with surgical clips or dynamic contrast-enhanced T1WI (DCE-T1WI). In addition, we also found that the time required for delineation with DE-MRI was obviously shorter than that with CT, which may be further helpful for radiation oncologists to improve their work efficiency and the accuracy of delineation in the clinic.

In order to ensure the efficacy of RT and avoid the radiotherapeutic toxicity, it is crucial to identify the target accurately. One of the main advantages of preoperative RT is identifying tumor site easier and delineating target volume better. Thus, the utilization of preoperative RT has been investigated and considered intriguing and of increasing interest [42]. Preoperative images, especially the common sequences of MRI, have been proved to be feasible in delineating the targets for preoperative RT [43]. The present study found DE-MR simulation images was feasible to contour the LC for prone RT. Accordingly, the new scanning sequence (breath holding- DE-MR) may be helpful to identify the tumor and improve the accuracy of target for preoperative RT. Given that all enrolled patients were rigorously screened, the sample of this study is a little small. Therefore, we will increase the number of suitable patients to further verify our result, and also further clarify the principle of how delayed time poses an effect on the LC defined by DE-T1WI in the future.

## Conclusions

For patients with a low SCS or an invisible seroma in the surgical cavity after BCS, it is reasonable to use prone DE-T1WI simulation scans to guide LC delineation. The LCs defined at 10 min postinjection with DE-T1W images offered modest coverage compared with the LCs defined with CT simulation images based on titanium surgical clips regardless of the volumes and locations of the LCs. Inter (intra) variability was minimal for the delineation of the LC based on 10th min DE-TIWI. DIR was used to minimize the spatial dislocation of targets caused by registration between CT and MR simulation images in this work. Prone simulation scans not only aid in LC delineation but also detect LCs located distant from the chest wall, thus avoiding the effect of an enhanced pectoralis on LC delineation.

## Data Availability

The datasets used and/or analyzed during the current study are available from the corresponding author on reasonable request.

## Abbreviations

DE-MR: Delayed-enhancement magnetic resonance
LC: Lumpectomy cavity
SCS: Seroma clarity score
BCS: Breast-conserving surgery
DIR: Deformable image registration
CT: Computed tomography
T2WI: T2-weighted imaging
DE-T1WI: Delayed-enhancement T1-weighted imaging
CI: Conformal index
DI: Degree of inclusion
COM: Center of mass
CIgen: Generalized conformity index
BCT: Breast-conserving therapy
RT: Radiotherapy
WBI: Whole breast irradiation
PBI: Partial breast irradiation
LRR: Locoregional recurrence
OS: Overall survival
MRI: Magnetic resonance imaging
DE-MRI: Delayed-enhancement magnetic resonance imaging
3D: Three-dimensional
LR: Lateral
AP: Anteroposterior
SI: Superoinferior
DCIS: Ductal carcinoma in situ
IDC: Invasive ductal carcinoma
DCE-T1WI: Dynamic contrast-enhanced T1-weighted imaging
